# Large-scale functional network connectivity mediate the associations of white matter lesions with executive functions and information processing speed in asymptomatic cerebral small vessels diseases

**DOI:** 10.1016/j.nicl.2025.103773

**Published:** 2025-03-21

**Authors:** Jing Chen, Weiwei lu, Zhangyang Wang, Mingfang Shi, Zhang Shi, Weibin Shi

**Affiliations:** a Department of Neurology, Zhongshan Hospital, Fudan University, Shanghai, China; b Department of Rehabilitation, Zhongshan Hospital, Fudan University, Shanghai, China; c Department of Radiology, Zhongshan Hospital, Fudan University, Shanghai, China; d Health Examination Center, Zhongshan Hospital, Fudan University, Shanghai, China

**Keywords:** Large-scale brain networks, Functional connectivity, White matter lesions, Functional magnetic resonance imaging, Executive functions, Information processing speed

## Abstract

**Objective:**

To examine the role of the large-scale functional network connectivity between white matter lesions (WMLs) and cognitive behaviors in patients of asymptomatic cerebral small vascular diseases (CSVD).

**Methods:**

The study sample consisted of 211 asymptomatic CSVD patients with WMLs. Large-scale internetwork and intranetwork functional connectivity (FC) were calculated using a combination of resting-state functional MRI data and independent component analysis. Neuropsychological tests involve cognitive functions were also measured. Then, potential correlations between WMLs, functional network connectivity and cognitive behaviors were tested. Mediation analysis was used to explore the role of functional network connectivity between WMLs and cognitive behaviors.

**Results:**

We successfully identified fourteen meaningful resting-state functional networks. Internetwork FC between dorsal sensorimotor network (dSMN) and right frontoparietal network (rFPN), dSMN and left frontoparietal network (lFPN), auditory network (AN) and posterior default network (pDMN), AN and executive control network (ECN), ECN and salience network (SN), dorsal attention network (DAN) and ECN were significant correlated with volumes of WMLs. Executive function were associated with internetwork FC between AN and pDMN, ECN and SN. Moreover, internetwork FC between AN and pDMN, ECN and SN mediated the relations of WMLs with executive function (for AN and pDMN, indirect effect: −0.0371, 95% CI: −0.0829 to −0.0073; for ECN and SN, indirect effect: −0.03191, 95% CI: −0.0807 to −0.0047). Moreover, left inferior parietal lobule in rFPN, right precentral gyrus in anterior default network (aDMN), right paracentral lobue in pDMN and left precunues in ECN were related to volumes of WMLs. There is a significant association of WMLs with intranetwork FC in left precunues, which could mediate the link between WMLs and information processing speed (indirect effect: −0.0437, 95% CI: −0.1055 to −0.0081).

**Conclusion:**

WMLs in asymptomatic CSVD patients may induce large-scale connectivity changes including the internetwork FC and intranetwork FC, which might further influence executive function and information processing speed.

## Highlights

• Found WMLs volumes significantly correlated with inter − network FC in multiple network pairs.
• Linked executive function to inter − network FC between AN − pDMN and ECN − SN, with mediation effects.
• Related specific brain regions in different networks to WMLs volumes.
• Associated WMLs with intranetwork FC in left precunues, mediating WMLs − info processing speed link.

## 1 Introduction

White matter lesions (WMLs) are commonly observed abnormalities in magnetic resonance imaging (MRI) scans of the brain in asymptomatic cerebral small vessels diseases (CSVD)(Jochems et al., 2022). An accumulating body of research indicated that these hyperintense signals on T2-weighted images have been associated with various cognitive impairments in CSVD (Dhana et al., 2022, Guo and Shi, 2022, Huang et al., 2022, Vergoossen et al., 2021), particularly in executive function and information processing speed (Liu et al., 2022, Vergoossen et al., 2021). Employing graph theory methods, these studies delved into the exact topological patterns of white matter connectivity disruption in CSVD, the contribution made by WMLs and the consequent influence on cognitive function, as well as the cognitive impairment brought about by distinct dynamic node characteristics (Liu et al., 2022, Vergoossen et al., 2021). However, the underlying mechanisms through which WMLs influence these cognitive functions remain poorly understood, and it may be not so easy to predict trends in cognitive function changes in the initial phase of the disease.

Various neuroimaging techniques, such as diffusion tensor imaging (DTI) and functional magnetic resonance imaging (fMRI), have been applied to investigate the brain structural and functional connectivity changes in CSVD with WMLs (Nozais et al., 2023, Quandt et al., 2020). Studies have shown that CSVD with WMLs are related to alterations in terms of the brain function or structure (Benson et al., 2018, Wang et al., 2020), which may play the part of potential mediators of the relevance between WMLs and cognitive behaviors (Brugulat-Serrat et al., 2020, Hoagey et al., 2021). In our previous research, we concentrated on the impacts of both white − matter integrity and gray − matter volume in mediating the relationships between WMLs and cognitive behaviors in asymptomatic CSVD patients. Our findings indicated that executive function and information processing speed were linearly correlated with the white matter integrity of specific white matter fibers. For instance, the right interior frontal − occipital fasciculus and the right anterior thalamic radiation played a role in mediating the relationship between the volume of WMLs and either executive function or information processing speed (Chen et al., 2023a, Chen et al., 2023b). Our previous study was unable to detect cognitive behaviors had any relevance to gray matter volume in CSVD patients with WMLs (Chen et al., 2023a, Chen et al., 2023b). In terms of brain function, few studies have investigated the properties of large-scale network functional connectivity, and how WMLs and the network functional connectivity affect cognitive behaviors in asymptomatic CSVD patients with WMLs (Kantarovich et al., 2022). Functional network connectivity is a measure of the interconnectivity and coordination between different brain regions. It reflects the flow of information and communication within the brain and plays a crucial role in cognitive processes.

**Fig. 1:**
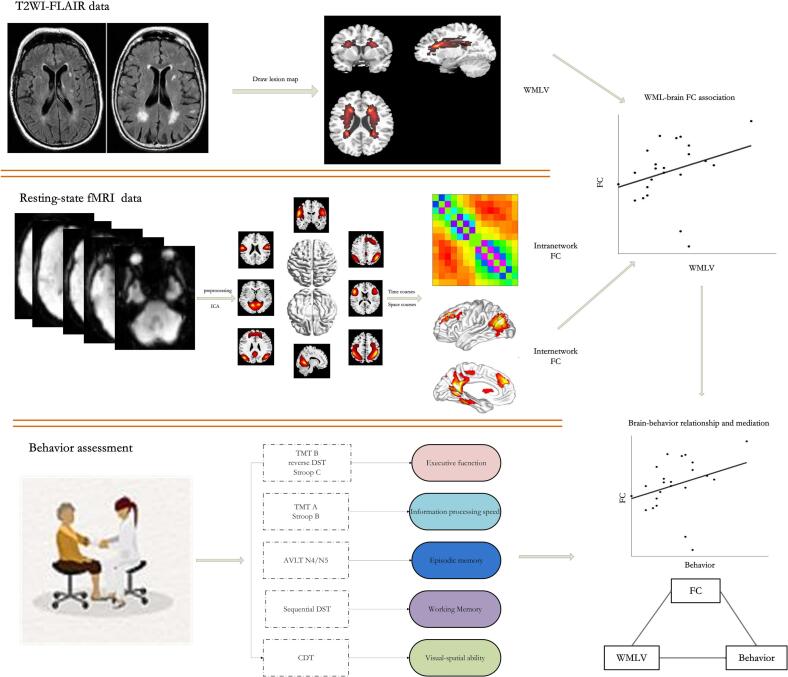
Flowchart of the study design. T2WI = T2-weighted images; FLAIR = fluid-attenuated inversion recovery images; WMLV = volume of white matter lesions; FC = functional connectivity; fMRI = functional magnetic resonance imaging; ICA = independent component analysis; TMT = trail-making test; DST = inverted digit span test; AVLT = auditory verbal learning test; and CDT = clock drawing test.

Independent component analysis (ICA) of resting-state fMRI data, regarded as a multivariate data-driven method, can identify and extract different resting-state networks, and further investigate internetwork and intranetwork functional connectivity (Calhoun et al., 2001, Calhoun et al., 2009). This method has been broadly used to explore the large-scale functional organization in normal and abnormal brains in the field of neuroscience and psychiatry (Barkhof et al., 2014, Jing et al., 2023, Xing et al., 2022). A recent study applied static and dynamic functional network connectivity methods to investigate the neural mechanism underlying WMH-related cognitive decrease in WMLs patients with mild cognitive disorders showed that intranetwork functional connectivity in visual network may mediate the decline of information processing speed caused by WMLs (Chen et al., 2023a, Chen et al., 2023b). However, up to now, little is known about the relationship between the large-scale internetwork and intranetwork functional connectivity and the WMLs in asymptomatic CSVD. Therefore, in this present study, we utilized a combination of resting − state functional MRI data and ICA to compute large scale internetwork and intranetwork functional connectivity. Our objectives were to explore whether the volumes of WMLs in asymptomatic CSVD patients have an impact on functional network connectivity, and to examine the role that large scale functional network connectivity plays in the relationship between WMLs and cognitive behaviors in these asymptomatic CSVD patients. The flowchart of study design is demonstrated in Fig. 1.Based on previous research results described above, we hypothesis that WMLs may induce large-scale internetwork FC and intranetwork FC connectivity changes, which might further influence executive function or information processing speed.

## 2 Materials and methods

### 2.1 Participants/Demographical information

This cross-sectional study was approved by the ethical council of Zhongshan Hospital Affiliated to Fudan University, and all participants provided written consent. This study included 1025 consecutive subjects recruited from the health examination center of Zhongshan Hospital Affiliated to Fudan University; of these, 814 subjects were excluded who did not meet the inclusion criteria or met the exclusion criteria, or who was with incomplete cognitive data, MRI data and excessive head motion during MRI scanning. Therefore, 211 subjects were used for analysis. Detailed information of the subjects is showed in Table 1. The inclusion criteria were as follows: (1) aged 50–85 years; (2) no overt clinical signsor positive symptoms of clinical manifestations upon neurophysical examination; (3) any degree of WMH changes in the subcortical or periventricular areas as seen on T2WI or FLAIR MRI; (4) being able to cooperate with MRI examination and neuropsychological scale tests; and (5) written informed consent. The exclusion criteria included: (1) aged 50 or 85 years; (2) other brain abnormalities or psychiatric diseases, or clinically significant or unstable medical diseases; (3) use of medications that might affect cognitive function, such as antipsychotics and antiepileptics; (4) leukoencephalopathy of nonvascular origin (immunological demyelinating, metabolic, toxic, infectious, other); and (5) contraindications for MRI scanning or claustrophobia.

**Table 1:** Demographics, clinical, cognitive and radiologic profiles of the study population.

	Total
No.	211
Age*, y	56.09 ± 7.80
Sex†, male	129 (61.1)
Educational level‡, y	9.00 [7.00 − 12.00]
BMI*, kg/m^2^	24.593 ± 3.23
Smoking†	96 (45.5)
Drinking†	100 (47.4)
Hypertension†	90 (42.7)
Diabetes mellitus†	30 (14.2)
Dyslipidemia†	112 (53.1)
MMSE*	28.28 ± 1.48
MoCA*	23.87 ± 3.53
Executive function‡	0.14 [-0.44 − 0.53]
Information processing speed‡	0.13 [-0.44 − 0.42]
Episodic memory‡	−0.04 [-0.41 − 0.70]
Working memory‡	0.17 [-0.53 − 0.88]
Visual-spatial ability‡	0.29 [-0.41 − 0.29]
Percentage normalized WMLs volume (×10^-4^) ^‡^	4.18 [2.10 − 8.68]
Mean FD	0.19 [0.13 − 0.31 ]

Abbreviations: BMI = body mass index, Mini-mental State Examination, MoCA = Montreal cognitive assessment, WMLs = white matter lesion, FD = framewise displacement.

* Data are showed as mean ± standard deviation.

† Data are showed as number of patients, with parentheses in percentages.

‡ Data are showed as median with interquartile range in brackets.

### 2.2 Cognitive assessment

For each participant, the neuropsychological measurements were meticulously arranged to be carried out on the same day as the brain MRI scan, all within one week following enrollment. The Mini Mental State Examination (MMSE) and Montreal Cognitive Assessment (MoCA) were measured as Global cognitive function. Each of the compound cognitive measure was acquired by converting the raw test results to Z scores. The average Z scores of the inverted digit span test, Stroop C and trail-Making test part B were applied to estimate executive function. The mean Z scores of the trail-making test part A and Stroop B were used to assess information processing speed. The mean Z scores of the auditory verbal learning test part N4 and N5 were applied to assess episodic memory; The Z score of sequential digit span test and clock drawing test were used to estimate working memory and visuospatial function, respectively.

### 2.3 MRI data acquisition

MRI data were acquired using a 3.0-Tesla GE Discovery MR750 system with a 32-channel head coil. Head motion was minimized by using tight but comfortable foam padding, and earplugs were worn to reduce scanner noise. High-resolution sagittal T1WI were obtained by a three-dimensional magnetization-prepared rapid gradient echo sequence: echo time (TE) = 3.1 ms, repetition time (TR) = 7.4 ms, field of view (FOV) = 256 mm × 256 mm, flip angle (FA) = 11°, matrix size = 256 × 256, voxel size = 1 mm × 1 mm × 1 mm, slice thickness = 1 mm, NEX = 1, and number of slices = 196. Resting-state BOLD fMRI data were obtained using a gradient-echo single-shot echo planar imaging (EPI) sequence with the following parameters: TE = 30 ms; TR = 2000 ms; FOV = 220 mm × 220 mm; FA = 90^0^; matrix size = 64 × 64; slice gap = 1 mm, slice thickness = 3 mm, numbers of volume = 200; and 43 interleaved axial slices. T2 fluid-attenuated inversion recovery images were obtained with a single shot fast spin echo sequence: TE = 97 ms, TR = 5500 ms, FOV = 256 mm × 256 mm, matrix = 256 × 256, slice thickness = 5 mm, number of slices = 22, and gap = 1 mm; All subjects were instructed to remain awake, keep their eyes closed, and attempt to think of nothing during scanning.

### 2.4 Wmls quantification

WMLs and the total brain volume were quantified using the semi-automated freeware NeuRoi (https://www.nottingham.ac.uk/research/groups/clinicalneurolo gy/neuroi.aspx). The WMLs lesions of each participant were meticulously outlined slice − by − slice in an independent manner by two neurologists, each having over eight years of professional experience. These two experts were kept completely unaware of other clinical data during the process. For the subsequent analyses, we utilized the average value of the total WMH volume calculated by these two researchers. The procedure can be seen in the previous published articles (Chen et al., 2023a, Chen et al., 2023b).

### 2.5 Resting-state fMRI data preprocessing

Resting-state fMRI data were preprocessed using Statistical Parametric Mapping (SPM12, https://www.fil.ion.ucl.ac.uk/spm) and Data Processing Assistant for Resting-State fMRI. The first 10 volumes from each subject were discarded to allow the signal to reach equilibrium and for the participants to adapt to the scanning noise. The remaining volumes were corrected for acquisition time delay between slices. Realignment was performed to correct the motion between time points. Any participants with head motion > 2.0 mm or in any direction > 2.0° were excluded. Individual functional images were normalized to the Montreal Neurological Institute (MNI) space and resampled into a voxel size of 3 × 3 × 3 mm^3^. After normalization, images were smoothed using a Gaussian kernel of 8 × 8 × 8 mm^3^ full-width at half-maximum.

### 2.6 Independent component analysis

**Fig. 2:**
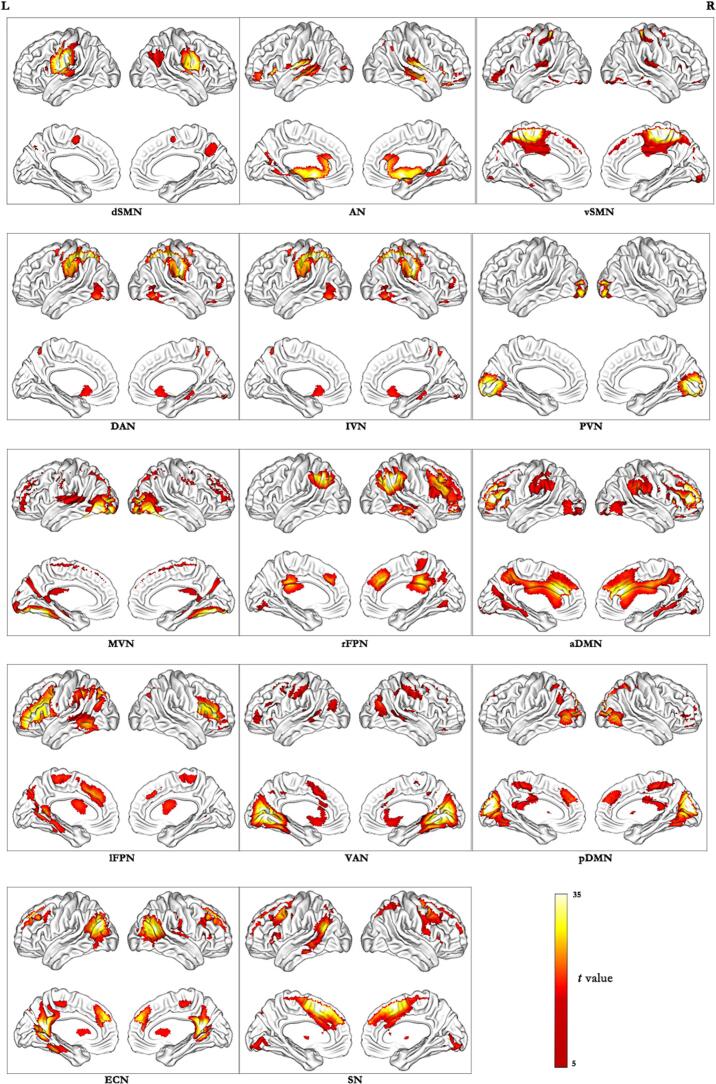
Cortical representation of the 14 selected resting state networks identified by independent component analysis. The color scale represents the *t* values in each functional network. dSMN = dorsal sensorimotor network; AN = auditory network; vSMN = ventral sensorimotor network; DAN = dorsal attention network; IVN = lateral visual network; pVN = primary visual network; mVN = medial visual network; rFPN = right frontoparietal network; aDMN = anterior default network; lFPN = left frontoparietal network; VAN = ventral attention network; pDMN = posterior default network; ECN = executive control network; and SN = salience network.

Maps of network connectivity were obtained by computing ICA using GIFT toolbox (mialab.mrn.org/software/gift/). There were 22 independent components been estimated automatically using the minimum description length criteria. The subject data were decomposed into linear mixtures of spatially independent components using spatial ICA, each of which exhibit a unique time course profile. Two data reduction steps were needed to achieve this. The first step is to reduce the subject-specific imaging data into 49 primary components using principal component analysis. Next, the infomax algorithm was used to decompose the reduced data of all participants into 22 estimated independent components. The infomax algorithm was performed 20 times in ICASSO (http:// research.ics.tkk.fi/ica/icasso/) to ensure the stability of the prediction, and polymerized spatial maps were estimated as the pattern of component clusters. Finally, each individual subject specific spatial maps and time courses were achieved using spatiotemporal regression back reconstruction method. Fourteen meaningful components were identified out of 22 independent components as resting-state networks via visual inspection (Fig. 2): dorsal sensorimotor network (dSMN), auditory network (AN), ventral sensorimotor network (vSMN), dorsal attention network (DAN), lateral visual network (lVN), primary visual network (pVN), medial visual network (mVN), right frontoparietal network (rFPN), anterior default network (aDMN), left frontoparietal network (lFPN), ventral attention network (VAN), posterior default network (pDMN), executive control network (ECN) and salience network (SN).

Functional connectivity between the identified 14 resting-state networks was computed using the Pearson correlation coefficients between pairs of time courses of the networks. Before this, the following processing procedures were performed: nuisance covariates (24 head motion parameters, cerebrospinal fluid signal, white matter signal and linear trend) were removed by regression, and the datasets were temporally band-pass filtered (0.01 to 0.08 Hz). Pearson correlation coefficients of the mean time courses between all pairs of resting-state networks were estimated for each participant and then converted to Z values using Fisher’s Z transformation. Intranetwork functional connectivity was estimated using the spatial maps, reflecting the degree of relevance of the time series of a given voxel to the average time series of its corresponding component.

### 2.7 Statistical analysis

The statistical descriptive analyses of demographic, volume of WMLs, cognitive behavioral and imaging data were carried out using the SPSS 24.0 software package (SPSS, Chicago, IL). We evaluated the normality of the data distribution through the Kolmogorov–Smirnov method and by examining the histogram. For continuous variables, the data are presented either as the mean and standard deviation or as the median and interquartile range. As for categorical variables, they are reported in terms of the number and percentage. For analysis of functional connections between networks, false discovery rate (FDR) with a corrected significance level of *p* < 0.05 was used for multiple comparisons. For analysis of functional connections within the network, all subjects' spatial maps for each functional network were firstly entered into a random-effect one-sample *t*-test to generate a sample-specific map. For each network, brain regions within the sample-specific map were considered if they met a height threshold of *p* < 0.05 corrected for multiple comparisons using a family-wise error (FWE) and an extent threshold of 100 voxels.

A multistep method was used to analyze the data of WMLs in asymptomatic CSVD, neuroimaging (large-scale internetwork and intranetwork functional connectivity), and cognitive behaviors (executive function, information processing speed, episodic memory, working memory and visuospatial function). First, partial correlation analysis was applied to test for the WMLs-brain associations between volume of WMLs and large-scale functional connectivity with age, sex, education level and mean FD as nuisance covariates. Then, for functional connectivity showing associations with volume of WMLs, correlations between these internetwork or intranetwork functional connectivity with cognitive behavioral variables were further explored using partial correlations adjusting for age, sex, education level and mean FD. Multiple comparisons were performed by FDR methods. Finally, mediation analysis was computed to further examine the role of functional connectivity in the relationship between WMLs and cognitive function using the PROCESS macro (https://www.processmacro.org/) installed in SPSS 24.0. Here, only variables that showed a significant correlation with others were considered independent (volume of WMLs), dependent (cognitive behaviors), or mediating (internetwork or intranetwork functional connectivity) variables in the mediation analysis. Sex, age, education level, smoking, drinking, BMI, hypertension, diabetes mellitus, dyslipidemia and mean FD were considered nuisance variables. The key estimates of interest were the magnitude of changes in the indirect path from volume of WMLs to cognition (a and b), and the direct path between volumes of WMLs and cognition (c in the bivariate models and c′ in the full mediation models). The significance analysis was based on 5000 bootstrap realizations and a significant indirect effect is considered when the bias-corrected 95 % confidence interval (CI) does not include zero.

## 3 Results

### 3.1 Associations between WMLs, internetwork functional connectivity between resting-state networks, and cognitive behaviors

**Fig. 3:**
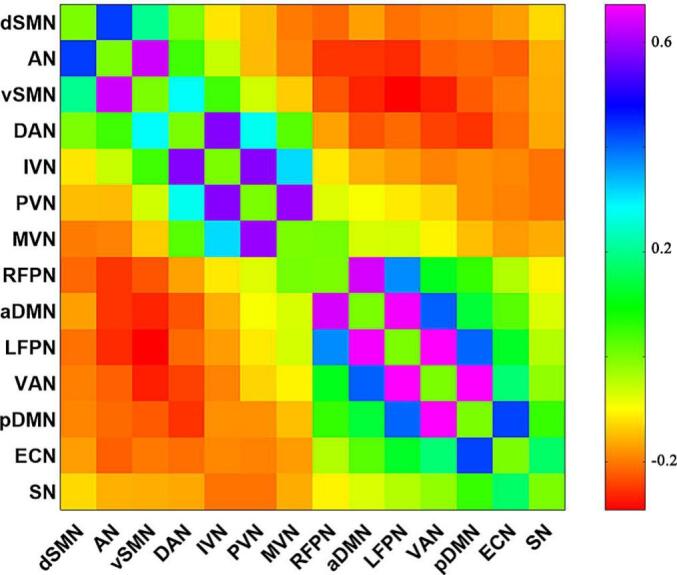
Internetwork functional connectivity matrix. Pairwise correlations between functional networks were averaged across subjects. Colors bar represent positive or negative functional connectivity. dSMN = dorsal sensorimotor network; AN = auditory network; vSMN = ventral sensorimotor network; DAN = dorsal attention network; IVN = lateral visual network; pVN = primary visual network; mVN = medial visual network; rFPN = right frontoparietal network; aDMN = anterior default network; lFPN = left frontoparietal network; VAN = ventral attention network; pDMN = posterior default network; ECN = executive control network; and SN = salience network.

**Fig. 4:**
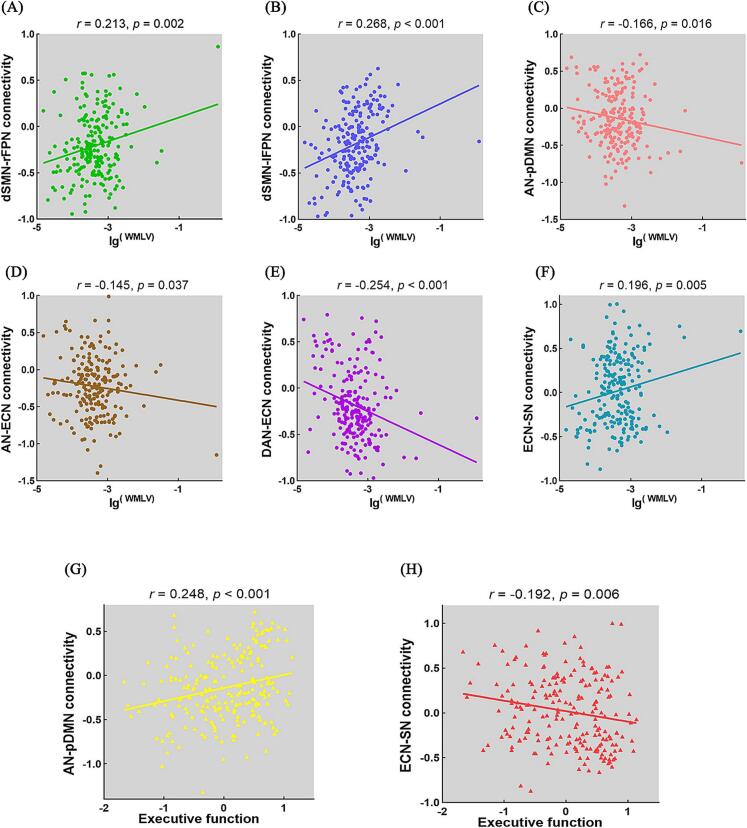
Correlations between internetwork functional connectivity and WMLs/behaviors. (A).between dSMN-rFPN FC and WMLs; (B).between dSMN-lFPN FC and WMLs; (C).between AN-pDMN FC and WMLs; (D). between AN-ECN FC and WMLs; (E).between DAN-ECN FC and WMLs; (F).between ECN-SN FC and WMLs; (G). between AN-pDMN FC and executive function; (I).between ECN-SN FC and executive function. WMLs = white matter lesions; FC = functional connectivity; dSMN = dorsal sensorimotor network; rFPN = right frontoparietal network; lFPN = left frontoparietal network; AN = auditory network; pDMN = posterior default network; ECN = executive control network; DAN = dorsal attention network; and SN = salience network.

Pairwise correlation patterns between large-scale functional networks are showed in Fig. 3. Both negative and positive large-scale functional connectivity between networks were identified. Relevance analyses showed significant associations between WMLs and internetwork functional connectivity (Fig. 4). Specifically, WMLs was positively associated with functional connectivity between dSMN and rFPN (*r* = 0.213, *p* = 0.002; Fig. 4A), between dSMN and lFPN (*r* = 0.268, *p* < 0.001; Fig. 4B), and between ECN and SN (*r* = 0.196, *p* = 0.005; Fig. 4F), and negatively associated with connectivity between AN and pDMN (*r* =  − 0.166, *p* = 0.016; Fig. 4C), between AN and ECN (*r* =  − 0.145, *p* = 0.037; Fig. 4D), and between DAN and ECN (*r* =  − 0.254, *p* < 0.001; Fig. 4E). Nevertheless, these differences were no longer significant after FDR correction.

**Fig. 5:**
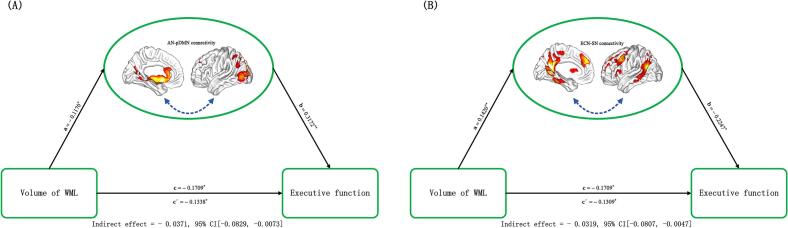
WMLs-internetwork functional connectivity-behaviors associations. (A) and (B) The mediation analyses between WMLs (X) and executive function (Y), with AN-pDMN and ECN-SN connectivity as the mediators (M). Path coefficients with *p* values (*p < 0.05 and **p < 0.01, respectively). AN = auditory network; pDMN = posterior default network; ECN = executive control network; and SN = salience network.

With regard to cognitive behaviors, executive function was observed to be positively related to AN-pDMN connectivity (*r* = 0.248, *p* < 0.001,uncorrected; Fig. 4G) and negatively related to ECN-SN connectivity (*r* =  − 0.192, *p* = 0.006,uncorrected; Fig. 4H). However, there were no significant relevance between internetwork functional connectivity and other cognitive functions. Moreover, mediation analyses showed that internetwork functional connectivity between AN-pDMN (indirect effect =  − 0.0371, standard error [SE] = 0.0187, 95 % CI: −0.0829, −0.0073) and ECN-SN (indirect effect =  − 0.0319, SE = 0.0189, 95 % CI: −0.0807, −0.0047) mediated the relationship between WMLs and executive function (Fig. 5A, B).

### 3.2 Associations between WMLs, intranetwork functional connectivity within resting-state networks, and cognitive behaviors

**Fig. 6:**
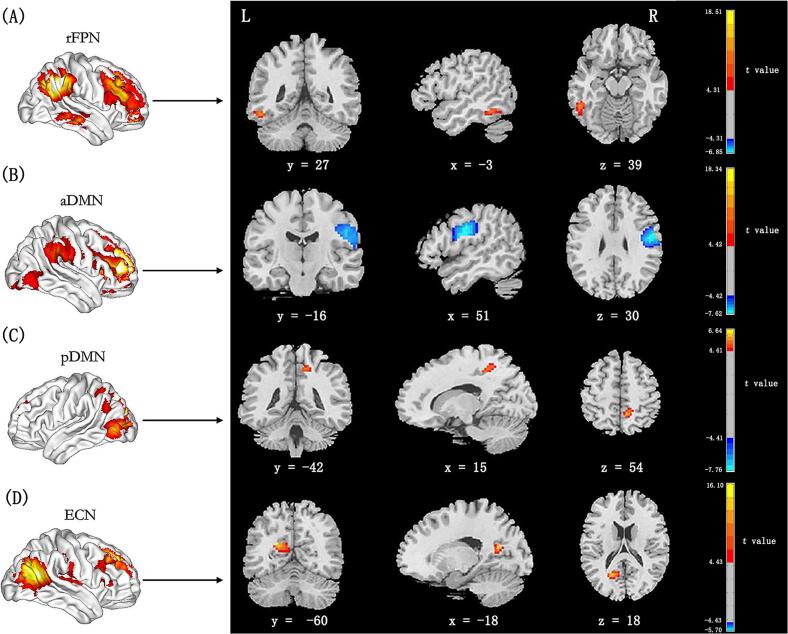
Correlations between WMLs and intranetwork functional connectivity. The color scale represents the *t* values in each network. Hot colors represent positive correlation and cool colors represent negative correlation. WMLs = white matter lesions; rFPN = right frontoparietal network; aDMN = anterior default network; pDMN = posterior default network; ECN = executive control network.

Large-scale functional connectivity intra network analyses revealed significant positive associations between WMLs and functional connectivity in left Inferior Parietal lobule (IPL) (peak MNI coordinate x/y/ z =  − 51/−48/−15, cluster size = 42 voxels, peak t = 5.45) of the rFPN (Fig. 6A), right Paracentral lobule (peak MNI coordinate x/y/z = 9/−48/54, cluster size = 76 voxels, peak t = 5.78) of the pDMN (Fig. 6C) and left Precunues (peak MNI coordinate x/y/z =  − 18/−60/18, cluster size = 55 voxels, peak t = 5.89) of the ECN (Fig. 6D), and a significant negative association between WMLs and functional connectivity in the right Precentral gyrus (peak MNI coordinate x/y/ z = 4/−33/35, cluster size = 41 voxels, peak t =  − 5.88) of the aDMN (Fig. 6B; *p* < 0.05, FDR corrected).

**Fig. 7:**
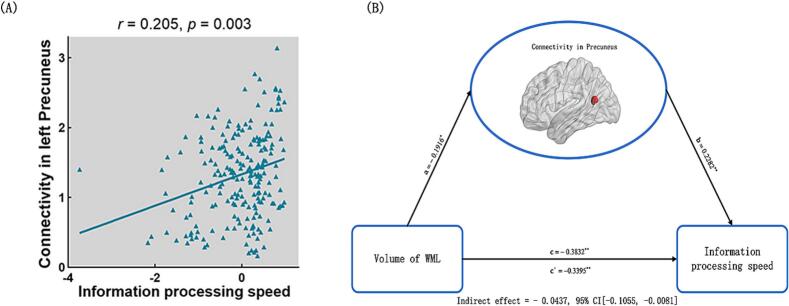
Associations between WMLs, intranetwork functional connectivity and behaviors. (A). The scatter plot shows the correlation between intranetwork functional connectivity in left Precuneus of ECN and information processing speed; (B) The mediation analysis between WMLs (X) and information processing speed (Y), with intranetwork connectivity in the left Precuneus as the mediator. Path coefficients with *p* values (*p < 0.05 and **p < 0.01, respectively). WMLs = white matter lesions.

With regard to cognitive behaviors, information process speed was found to be positively associated function connectivity in left Precunues (*r* = 0.205, *p* = 0.003, uncorrected; Fig. 7A). No significant relevance was found between other intranetwork functional connectivity and cognitive behavioral variables. Mediation analyses revealed that intranetwork functionl connectivity in left Precunues (indirect effect =  − 0.0437, standard error [SE] = 0.0238, 95 % CI: −0.1055, −0.0081) mediated the relationship between WMLs and information process speed (Fig. 7B).

## 4 Discussion

In the study, we found significant correlations of WMLs with functional connectivity between large-scale functional networks among the executive control (rFPN, lFPN, ECN, SN and DAN), default mode (aDMN and pDMN), and sensorimotor (dSMN and AN) systems before FDR correction, implying widespread but non-specific impacts of WMLs on internetwork functional connectivity. Additionally, executive function was associated with some of the WMLs-sensitive functional connectivity between networks, which could play the part of mediators of the correlation between WMLs and executive function. Moreover, WMLs revealed associations with intranetwork connectivity of the rFPN, aDMN, pDMN and ECN. Further, it was found that information processing speed was positively correlated with intranetwork functional connectivity in left Precunues of the ECN, which be able to act as the mediators of the correlation between WMLs and information processing speed. These results indicate pronounced and specific influences of WMLs in patients of asymptomatic CSVD on intranetwork connectivity within the executive control and default mode system.

Previous studies have made efforts to investigate the relationship between WMLs and neuroimaging measures in CSVD (Acharya et al., 2019, Kantarovich et al., 2022, Wu et al., 2023, Yu et al., 2023, Zanon et al., 2022). As we’ve seen, there is only one resting-state fMRI study attempted to explore the relevance between WMLs and large-scale internetwork and intranetwork functional connectivity in patients of asymptomatic CSVD. Results from the previous study demonstrated that higher load of WMLs was correlated with a universal mode of network partitioning, charactered by greater internetwork and lower intranetwork connectivity (Kantarovich et al., 2022). Also, the study showed that the relationship between WMLs and functional connectivity was negatively correlated with cognitive abilities in healthy older adults with WMLs ((Kantarovich et al., 2022). In that study, the large scale networks were constructed based on the well − defined functional networks in the atlas. However, this approach might lack an independent and comprehensive perspective. Compared with data-driven ICA, this may result in potential selection bias that might have an impact on research results. Consequently, there remains a need for studies to objectively elucidate the impacts of WMLs on large − scale functional networks and cognitive behaviors. Moreover, consistent evidence has demonstrated that the correlations between WMLs and cognitive behaviors including executive function, information processing speed and working memory in CSVD (Beck et al., 2021, Kerkhofs et al., 2021, Puzo et al., 2019). In the present study, with the availability of data of volume of WMLs, a set of refined cognitive behaviors assessments and resting-state fMRI data makes it possible to further uncover the relationships of WMLs-functional network connectivity-behavior in patients of asymptomatic CSVD.

The executive control system, mainly referring to the prefrontal cortex and lateral parietal regions, is considered to be involved in a series of cognitive-control processes including self-regulation, control of cognition, temporal organization of response to immediate stimuli, planning behavior, and control of attention, via a complex and coordinated operation of multiple networks (Ren et al., 2020, Cole and Schneider, 2007, Breukelaar et al., 2017). The default mode system primarily includes the medial temporal lobe, the medial prefrontal cortex, the posterior cingulate cortex, the ventral precuneus and parts of the parietal cortex. These areas were correlated with some aspect of internal thought, such as memory, emotional processing, theory of mind, self referential mental activity and integrating different kinds of internal thoughts (Andrews-Hanna et al., 2010, Fair et al., 2008).^31,32^ The observed strong effects of WMLs on functional connectivity between network and within network of the executive control and default mode system highlight its prominent role in cognitive behaviors. Meanwhile, the present study found that functional connectivity of the sensorimotor system was also affected by the WMLs. The sensorimotor system primarily consists of premotor cortex, primary motor cortex, supplementary motor area, frontal-parietal lobe and other subcortical structures, which is implicated in a range of motor and sensory processes (Goelman et al., 2023, Kleinfeld and Deschenes, 2011). Our results was in line with that from previous researches that reported correlations between WMLs in CSVD and resting-state functional connectivity of the sensorimotor systems (Camicioli and Verghese, 2015, Wang et al., 2023). The effects of WMLs on the above three system highlight that human cognitive behavior is a whole, via complicated interactions between the critical networks (functional connectivity within network) and with other components (functional connectivity between network).

In the present study, the mediation analyses further showed that large-scale functional network connectivity mediated the associations of WMLs with executive function and information processing speed, which are crucial for cognitive performance and daily activities. Our study has important clinical implications. Understanding the mechanisms linking WMLs to cognitive behaviors could help develop targeted interventions for improving cognitive performance in individuals with white matter lesions. For example, interventions aimed at enhancing functional network connectivity through cognitive training or neuromodulation could prove beneficial for improving cognitive functions in this population.

Several limitations should take into consideration in the present study. First, as the cross-sectional natural of the design, the longitudinal data will be required to further clarify the relationship and mediating mechanism between WMLs and cognitive behaviors. Second, we focused on the effects of WMLs on cognitive behavior, but other age-related neurobiological changes (e.g., grey matter loss) should be calculated to further examine their impact on functional connectivity. Third, the mean age of the population encompassed in this study is 56.09 years, which is relatively young. Therefore, when applying the conclusions of this study to the elderly population, we should be prudence. Finally, although meaningful functional networks were acquired from a series of ICA-derived components on the basis of a strict selection procedure, there may be some biases that have affected our results.

Our findings suggest that large-scale functional network connectivity mediates the relationships between white matter lesions and executive functions/information processing speed. This finding may add to our understanding of the complex associations between white matter integrity, functional brain networks, and cognitive performance. Future studies could build on this work by investigating the potential for targeted interventions aimed at improving cognitive functions in asymptomatic CSVD individuals with WMLs.

## CRediT authorship contribution statement

**Jing Chen:** Writing – review & editing, Writing – original draft, Funding acquisition, Conceptualization. **Weiwei lu:** Software, Methodology, Investigation. **Zhangyang Wang:** Methodology, Investigation. **Mingfang Shi:** Resources, Project administration, Methodology, Investigation. **Zhang Shi:** Validation, Software, Resources, Project administration. **Weibin Shi:** Validation, Supervision.

## Declaration of Competing Interest

The authors declare that they have no known competing financial interests or personal relationships that could have appeared to influence the work reported in this paper.

## Data Availability

Data will be made available on request.
